# Web-based application for predicting the potential target phenotype for recombinant human thrombomodulin therapy in patients with sepsis: analysis of three multicentre registries

**DOI:** 10.1186/s13054-022-04020-1

**Published:** 2022-05-19

**Authors:** Tadahiro Goto, Daisuke Kudo, Ryo Uchimido, Mineji Hayakawa, Kazuma Yamakawa, Toshikazu Abe, Atsushi Shiraishi, Shigeki Kushimoto

**Affiliations:** 1 Department of Clinical Epidemiology and Health Economics, School of Public Health, The University of Tokyo, Tokyo, Japan; 2 TXP Medical Co. Ltd., 7-3-1 Hongo, Bunkyo-ku, Tokyo, 113-0033 Japan; 3 Division of Emergency and Critical Care Medicine, Tohoku University Graduate School of Medicine, Sendai, Japan; 4 Department of Intensive Care Medicine, Tokyo Medical and Dental University, Tokyo, Japan; 5 Department of Emergency Medicine, Hokkaido University Hospital, Sapporo, Japan; 6 Department of Emergency Medicine, Osaka Medical and Pharmaceutical University, Osaka, Japan; 7 Department of Emergency and Critical Care Medicine, Tsukuba Memorial Hospital, Tsukuba, Japan; 8 Health Services Research and Development Center, University of Tsukuba, Ibaraki, Japan; 9 Emergency and Trauma Center, Kameda Medical Center, Chiba, Japan

**Keywords:** Recombinant human thrombomodulin, Sepsis, Phenotype, Prediction model, Coagulopathy

## Abstract

A recent randomised controlled trial failed to demonstrate a beneficial effect of recombinant human thrombomodulin (rhTM) on sepsis. However, there is still controversy in the effects of rhTM for sepsis due to the heterogeneity of the study population. We previously identified patients with a distinct phenotype that could be a potential target of rhTM therapy (rhTM target phenotype). However, for application in the clinical setting, a simple tool for determining this target is necessary. Thus, using three multicentre sepsis registries, we aimed to develop and validate a machine learning model for predicting presence of the target phenotype that we previously identified for targeted rhTM therapy. The predictors were platelet count, PT-INR, fibrinogen, fibrinogen/fibrin degradation products, and D-dimer. We also implemented the model as a web-based application. Two of the three registries were used for model development (*n* = 3694), and the remaining registry was used for validation (*n* = 1184). Approximately 8–9% of patients had the rhTM target phenotype in each cohort. In the validation, the C statistic of the developed model for predicting the rhTM target phenotype was 0.996 (95% CI 0.993–0.998), with a sensitivity of 0.991 and a specificity of 0.967. Among patients who were predicted to have the potential target phenotype (predicted target patients) in the validation cohort (*n* = 142), rhTM use was associated with a lower in-hospital mortality (adjusted risk difference, − 31.3% [− 53.5 to − 9.1%]). The developed model was able to accurately predict the rhTM target phenotype. The model, which is available as a web-based application, could profoundly benefit clinicians and researchers investigating the heterogeneity in the treatment effects of rhTM and its mechanisms.

**Supplementary Information:**

The online version contains supplementary material available at 10.1186/s13054-022-04020-1.

## Background

Recombinant human thrombomodulin (rhTM) has been suggested as an adjunct therapy for patients with sepsis [1]. A recent randomised controlled trial (RCT) failed to demonstrate its beneficial effect on 28-day mortality [2], but there remains controversy in the results of this study due to the heterogeneity of its study population. Indeed, 22% of patients in the RCT did not meet protocol-specified coagulopathy. In addition, an updated meta-analysis including the RCT reported an association between rhTM use and a lower risk of mortality [3]. These findings collectively suggest the importance of appropriately targeting the study population prior to conducting studies to gain maximum benefit [4–6]. We identified a distinct phenotype that could be a potential target of rhTM therapy [7], a finding consistent with previously suggested targets, including coagulation disorder and high disease severity [8, 9]. However, for application in the clinical setting, a simple tool for determining this target is necessary [10]. Thus, we aimed to develop and validate a model for predicting the potential target phenotype for rhTM therapy and to implement the model as a web-based application to facilitate further research.

## Methods

### Study design and settings

**Fig. 1:**
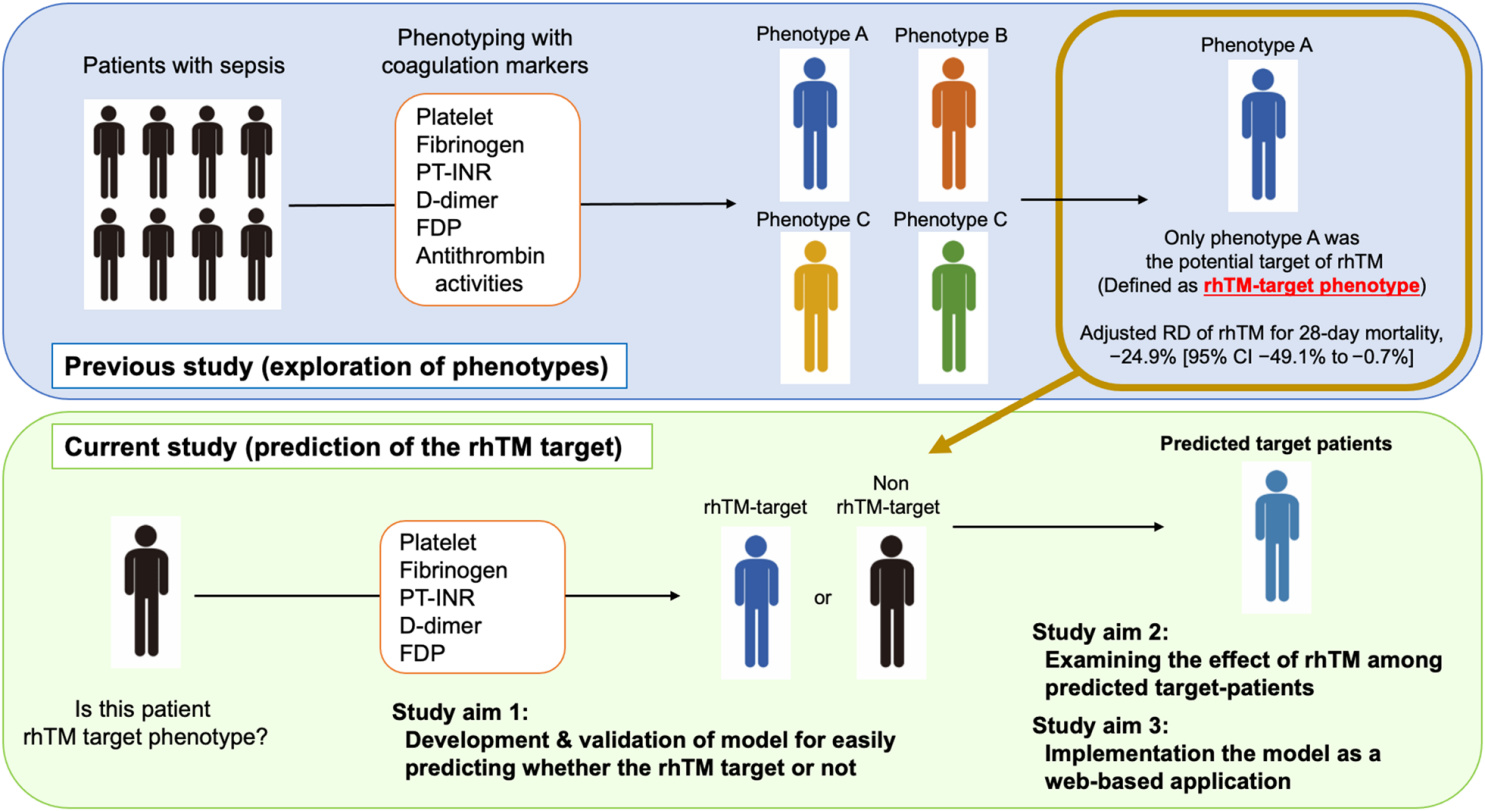
Current study (development and implementation of a prediction model of rhTM target phenotype)

The concept of this study is shown in Fig. 1. Details of this study are provided in Additional file 1. This was a secondary analysis of the following multicentre registries: the Japan Septic Disseminated Intravascular Coagulation (JSEPTIC-DIC) study (42 ICUs at 40 institutions, 2011–2013) [11], Tohoku Sepsis Registry (10 institutions, 2015) [12], and Focused Outcomes Research in Emergency Care for Acute Respiratory Distress Syndrome, Sepsis, and Trauma (FORECAST) sepsis study (59 ICUs, 2016–2017) [13]. These studies were approved by the institutional review boards at the participating hospitals, and the need for informed consent was waived.

### Study samples

We included all patients (aged ≥ 16 years), who were admitted to the ICU with severe sepsis or septic shock as defined in the three registries, according to *the International Sepsis Definitions Conference Criteria* [14, 15]. We excluded patients with missing information on 28-day mortality, which is required for determining the phenotype of each patient [7].

### Predictors

We used the following coagulation markers for predicting the presence of the target phenotype, in accordance with our previous study [7]: platelet counts, PT-INR, fibrinogen, fibrinogen/fibrin degradation products (FDP), and D-dimer.

### Outcomes

The primary outcome was the presence of the clinical phenotype identified in our previous study [7], characterised as severe physiological status and organ dysfunction (high Acute Physiology and Chronic Health Evaluation [APACHE II] and Sequential Organ Failure Assessment [SOFA] scores), coagulopathy (low platelet count, prolonged PT-INR, low fibrinogen, and extremely high FDP and D-dimer levels), high lactate level, and high mortality. We termed this phenotype as “rhTM target phenotype”.

### Statistical analysis

We derived our prediction model using the JSEPTIC-DIC study and Tohoku Sepsis Registry (derivation cohort) and validated the model using the FORECAST sepsis study (validation cohort). We imputed missing predictors using the random forest method with the *missForest* package (Additional file 2: Table S1) [16]. We did not calculate the sample size in advance because we used all available data. The sample size for model development (*n* = 3694, of which 9% had the target phenotype) was enough to ensure precise predictions and minimise overfitting [17].

For model development, we divided the derivation cohort into the training set (70% of the full sample randomly chosen for model development and hyperparameter tuning) and test set (30% of the full sample randomly chosen for internal validation). Using the training set, using log-transformed predictors, we constructed a prediction model with XGBoost. We used the grid search strategy to identify the best combination of hyperparameters using the *ranger* and *caret* packages with tenfold cross validation.

We measured the prediction performance of the developed model by computing the (1) C statistic (i.e., the area under the receiver operating characteristic [ROC] curve) and (2) prospective prediction results.

In addition, among patients those who were predicted to have the potential target phenotype (termed as “predicted target patients”), we assessed the effect of rhTM on in-hospital and 28-day mortality using a generalised estimating equation to account for patient clustering within hospitals. The adjusted variables were selected according to the previous study [7] (see Additional file 1). We also reported the number of patients who met the inclusion criteria for the SCARLET trial (cardiovascular and/or respiratory dysfunction, and PT-INR > 1.4 and a platelet count in the range from 30 to 150 × 10^9^/L) to illustrate the difference in the target study population between studies [2].

Lastly, we uploaded the model online (URL: http://research-kudo-prediction.s3-website-ap-northeast-1.amazonaws.com/), so researchers interested in utilising the model could access it for free. All analyses were performed with R statistical software version 3.6.1 (R Foundation for Statistical Computing).

## Results

Patient characteristics were similar between predicted target patients and patients with rhTM target phenotype (Table 1). However, predicted target patients in the current study were likely to have milder coagulopathy. Approximately 8–9% of patients had the rhTM target phenotype. Overall, patients who met the inclusion criteria for the SCARLET trial accounted for 20–30% of rhTM target phenotype.

**Table 1:** Characteristics and clinical course of patients with sepsis in the derivation and validation cohorts

Variables	Test set of the derivation cohort (*n* = 1108)		Validation cohort (*n* = 1184)	
	Predicted target patients *n* = 118	Patients with rhTM target phenotype *n* = 85	Predicted target patients *n* = 142	Patients with rhTM target phenotype *n* = 108
Age, median (IQR)	71 (56, 79)	70 (55, 79)	73 (64, 82)	73 (64, 82)
Sex, female	60 (51%)	42 (49%)	61 (43%)	45 (42%)
Body weight (kg), median (IQR)	54 (49, 63)	55 (49, 64)	55.0 (47.0, 65.0)	53.0 (46.5, 60.5)
*Infection site*				
Catheter-related	2 (2%)	0 (0%)	7 (5%)	6 (6%)
Bone/soft tissue	10 (8%)	7 (8%)	11 (8%)	7 (6%)
Cardiovascular	5 (4%)	5 (6%)	5 (4%)	5 (5%)
Central nervous system	3 (3%)	3 (4%)	2 (1%)	2 (2%)
Urinary tract	28 (24%)	21 (25%)	39 (27%)	31 (29%)
Lung/thoracic	16 (14%)	13 (15%)	24 (17%)	19 (18%)
Abdomen	33 (28%)	19 (22%)	38 (27%)	25 (23%)
Other/unknown	21 (18%)	17 (20%)	16 (11%)	13 (20%)
APACHE II, median (IQR)	28 (21, 34)	27 (21, 34)	27 (22, 33)	27 (22, 32)
SIRS score, median (IQR)	3 (3, 4)	3 (3, 4)	3 (3, 4)	3 (3, 4)
SOFA scores	13 (10, 16)	13 (10, 16)	13 (11, 13)	11 (9, 14)
*Lab data*				
White blood cell (10^3^/μL), median (IQR)	11.5 (2.7, 20.3)	12.4 (2.7, 20.7)	11.0 (6.1, 20.1)	10.8 (6.3, 20.0)
Platelet (10^3^/μL), median (IQR)	54 (29, 99)	47 (26, 76)	73 (42, 122)	68 (41, 121)
PT-INR, median (IQR)	1.7 (1.4, 2.1)	1.7 (1.4, 2.1)	1.5 (1.3, 1.7)	1.5 (1.3, 1.7)
Fibrinogen (mg/mL), median (IQR)	237 (141, 328)	220 (130, 311)	269 (153, 378)	277 (154, 381)
FDP (μg/mL), median (IQR)	98 (68, 224)	127 (80, 299)	107 (73, 188)	121 (93, 245)
D-dimer (μg/mL), median (IQR)	42 (31, 94)	51 (34, 119)	48 (32, 83)	60 (40, 106)
Antithrombin (%), median (IQR)	51 (42, 60)	50 (42, 58)	54 (48, 65)	55 (49, 66)
Lactate (mmol/L), median (IQR)	6 (3, 10)	6 (4, 10)	5 (3, 7)	5 (3, 7)
Patients who met the inclusion criteria for the SCARLET trial*				
Coagulopathy	29 (25%)	23 (27%)	32 (23%)	33 (31%)
Coagulopathy and respiratory/cardiovascular dysfunction	25 (21%)	21 (25%)	27 (19%)	20 (16%)
*Management*				
rhTM	52 (44%)	41 (48%)	55 (39%)	44 (44%)
Vasopressor use	105 (89%)	77 (91%)	103 (73%)	77 (71%)
Renal replacement therapy	51 (43%)	40 (47%)	23 (16%)	16 (15%)
Steroids	35 (30%)	26 (31%)	65 (48%)	48 (44%)
Intravenous immunoglobulin	58 (49%)	40 (47%)	50 (35%)	18 (17%)
Antithrombin	59 (50%)	41 (48%)	28 (20%)	22 (20%)
*Prognosis*				
28-day death	48 (41%)	36 (42%)	40 (28%)	24 (25%)
In-hospital death	62 (53%)	47 (55%)	47 (33%)	28 (28%)

APACHE, Acute Physiology and Chronic Health Evaluation; FDP, fibrinogen/fibrin degradation product; IQR, interquartile range; PT-INR, prothrombin time-international normalised ratio; SIRS, systemic inflammatory response syndrome; SOFA, Sequential Organ Failure Assessment; and WBC, white blood cells

Five coagulation markers (in bold) were used for prediction

*Defined as patients with (1) coagulopathy (PT-INR > 1.4 and platelet count 30 to 150 × 10^9^/L) and (2) vasopressor use or mechanical ventilation use

**Fig. 2:**
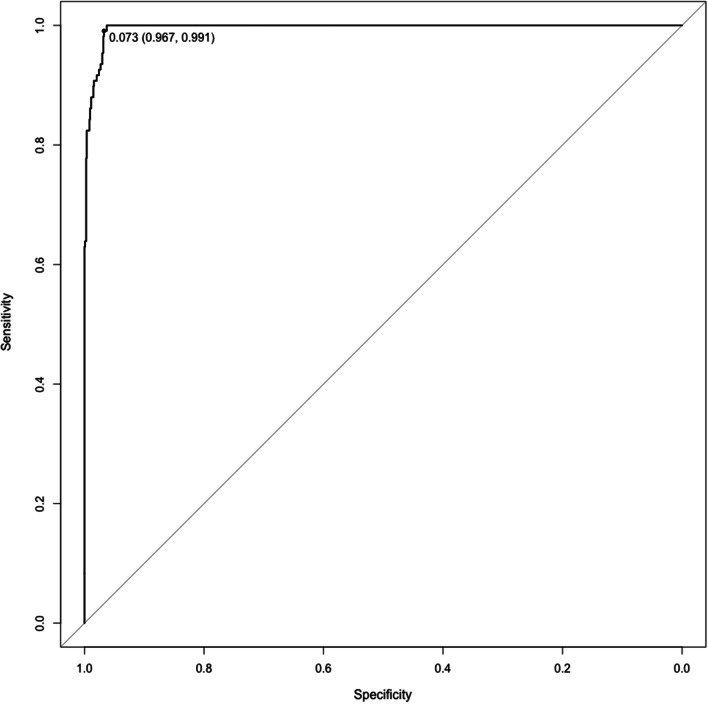
The receiver operating characteristic curve of the developed model for predicting the presence of the target phenotype in the external validation cohort

Using the test set of the derivation cohort, we found that the C statistic of the developed model was 0.993 (95% CI 0.989–0.997). Prospective prediction results were as follows: sensitivity 0.968, specificity 0.955, positive predictive value 0.669, and negative predictive value 0.997. Figure 2 shows the prediction ability of the developed model in the validation cohort. Using the validation cohort, we found that the model had high discrimination (C statistic, 0.996; 95% CI 0.993–0.998). Prospective prediction results were as follows: sensitivity 0.991, specificity 0.967, positive predictive value 0.754, and negative predictive value 0.999.

Among predicted target patients in the validation cohort, rhTM use was associated with a lower in-hospital mortality (adjusted risk difference, − 31.3% [− 53.5 to − 9.1%]; Table 2).

**Table 2:** Unadjusted and adjusted risk difference between recombinant thrombomodulin use and outcomes among predicted target patients

Predicted target patients	In-hospital mortality		28-day mortality	
	Unadjusted risk Difference (95% CI)	Adjusted risk difference (95% CI)	Unadjusted risk Difference (95% CI)	Adjusted risk difference (95% CI)
Test set of the derivation cohort (*n* = 118)	− 22.0% (− 40.6 to − 3.4%)	− 27.4% (− 41.8 to − 12.9%)	− 20.0% (− 38.2 to − 1.8%)	− 23.6% (− 39.8 to − 7.4%)
Validation cohort (*n* = 142)	− 15.1% (− 31.1 to 1.0%)	− 31.3% (− 53.5 to − 9.1%)	− 8.4% (− 24.7 to 8.0%)	− 21.1% (− 43.4 to 1.1%)

In the test set of derivation cohort, the adjusted variables were age, sex, comorbidities, and Sequential Organ Failure Assessment (SOFA) scores

In the validation cohort, the adjusted variables were age, sex, comorbidities, SOFA scores, and in-hospital management, including renal replacement therapy, and treatment with steroids, intravenous immunoglobulin, antithrombin, and vasopressors

## Discussion

We derived and validated a machine learning model that accurately predicts the rhTM target phenotype in patients with sepsis and released it online for clinical and research use. The C statistic was 0.994 in the validation cohort, with a sensitivity of 0.981 and a specificity of 0.944. The predicted target patients were likely to have milder coagulopathy compared to those with rhTM target phenotype.

The importance of considering the heterogeneity in the study population and the treatment effects has been emphasised in recent years [6]. As shown in the analysis of multiple sepsis registries and RCTs [5], clinical phenotypes were correlated with host-response patterns and clinical outcomes, and simulations suggested the presence of heterogeneity in treatment effects across phenotypes. Thus, such heterogeneity may at least partially explain the underlying mechanisms of RCTs that failed to reveal significant benefit of therapies in critical care [18, 19]. Indeed, patients who met the inclusion criteria for the SCARLET trial accounted for 20–30% of the patients with rhTM target phenotype, suggesting that further studies are needed to investigate the effects of rhTM for sepsis. Additionally, the process of identifying the target population to be treated is important and should be discussed in future cost–benefit analyses of treatment strategies, even if a small proportion of patients can be treated effectively (as was the case in our study sample).

Subgroup analyses have been widely used to address treatment effect heterogeneity despite its limitations [20]. In particular, conventional subgroup analyses assess one characteristic at a time, which may not reflect the biology or clinical practice where multiple factors often act synergistically [6]. To address this concern, several approaches have been proposed: clustering algorithms to identify distinct clinical phenotypes, Bayesian hierarchical models, and adaptive enrichment [6]. Building on these works, we used a clustering approach in a previous study to address the heterogeneity in our study population. While it is still challenging to find the *true* phenotypes that are responsible for the heterogeneity, we believe that our research process: (1) discovering the target phenotype, (2) implementing a model for predicting the phenotype, and (3) conducting studies for identifying the optimal target population or exploring underlying mechanisms—is an efficient way of conducting future studies and advancing personalised medicine. For example, our findings support the findings from a post hoc analysis of the SCARLET trial that reported an association between higher baseline thrombin generation biomarker levels and the effect of rhTM [9], by demonstrating that a subtype consisting of a high-dimensional coagulation profile could be a potential target of rhTM.

This study has several limitations. First, although we developed a model to predict the rhTM target phenotype, it remains unclear whether the rhTM target phenotype is the *true* target of rhTM therapy. Second, there may be diagnostic suspicion bias and unmeasured confounding. Additionally, the number of missing variables for prediction may have limited our findings. Thus, our findings should be validated in randomised controlled trials. Third, because machine learning models are generally difficult to interpret, our model itself does not provide information on the underlying mechanisms. Finally, our data were obtained from Japanese patients, and the generalisability of the results to other populations may be limited.

## Conclusions

We developed a model that accurately predicted the rhTM target phenotype. Our model is available online, which could profoundly benefit clinicians and researchers investigating the heterogeneity in the treatment effects of rhTM and its mechanisms.

## Supplementary Information

**Additional file 1**. Methods.

**Additional file 2: Table S1**. Missingness in predictors and outcome variables.

## Data Availability

JSEPTIC-DIC data are publicly available (*Sci Data*. 2018;5:180243). Tohoku Sepsis Registry and FORECAST sepsis study are not publicly available because participants of this study did not agree that their data can be shared publicly.

## Abbreviations

APACHE: Acute Physiology and Chronic Health Evaluation
FDP: Fibrinogen/fibrin degradation product
FORECAST: Focused Outcomes Research in Emergency Care for Acute Respiratory Distress Syndrome, Sepsis, and Trauma
ICU: Intensive care unit
JSEPTIC-DIC: Japan Septic Disseminated Intravascular Coagulation
PT-INR: Prothrombin time/international normalised ratio
RCT: Randomised controlled trial
rhTM: Recombinant human thrombomodulin
ROC: Receiver operating characteristic
SOFA: Sequential Organ Failure Assessment
