# Supplementary material for: Web-based application for predicting the potential target phenotype for recombinant human thrombomodulin therapy in patients with sepsis: analysis of three multicentre registries
Source: Crit Care. 2022 May 19;26:145. doi: 10.1186/s13054-022-04020-1 (PMC9121613; doi:10.1186/s13054-022-04020-1)
Supplement: Supplementary file 1 — Additional file 1. Methods. [file 13054_2022_4020_MOESM1_ESM.docx]

**ADDITIONAL FILE 1: METHODS**

**Web-based application for predicting potential target phenotype for recombinant human thrombomodulin therapy in patients with sepsis: analysis of three multicentre registries**

**METHODS**

*Study design and settings*

This study followed the Transparent Reporting of a Multivariable Prediction Model for Individual Prognosis or Diagnosis (TRIPOD) reporting guideline for prognostic studies [1]. This was a secondary analysis of the following multicentre registries: the Japan Septic Disseminated Intravascular Coagulation (JSEPTIC-DIC) study, Tohoku Sepsis Registry, and Focused Outcomes Research in Emergency Care for Acute Respiratory Distress Syndrome, Sepsis, and Trauma (FORECAST) sepsis study. All three registries include information on consecutive patients admitted to intensive care units (ICUs) for severe sepsis or septic shock. The JSEPTIC-DIC study retrospectively reviewed data derived from 3195 consecutive patients with severe sepsis or septic shock, aged ≥ 16 years, admitted to 42 ICUs at 40 institutions in Japan, between January 2011 and December 2013 [2]. The Tohoku Sepsis Registry prospectively registered 616 consecutive patients who were admitted to ICUs with severe sepsis, or those who developed severe sepsis after admission to the ICUs or general wards at 10 institutions (three university hospitals and seven community hospitals) in the Tohoku District, Northern Japan between January and December 2015 [3]. The multicentre prospective FORECAST sepsis study included 1184 consecutive patients aged ≥ 16 years, who were admitted to 59 ICUs in Japan with severe sepsis according to the sepsis-2 criteria between January 2016 and March 2017 [4]. These studies were approved by the institutional review boards at the participating hospitals and the need for informed consent was waived.

*Study samples*

We included all patients (aged ≥ 16 years), who were admitted to the ICUs with severe sepsis or septic shock as defined in the three registries, according to *the International Sepsis Definitions Conference criteria* [5, 6]. We excluded patients without information on 28-day mortality available, which is required for [7].

*Predictors*

We used the following coagulation markers for predicting the presence of the target phenotype according to our previous study [7]: platelet counts, PT-INR, fibrinogen, fibrinogen/fibrin degradation products (FDP), and D-dimer. We excluded antithrombin activities from the predictors because of its large proportion of missing data (52% and 47% of the data were missing in the derivation and validation cohorts, respectively).

*Outcomes*

The primary outcome was the presence of the clinical phenotype identified in our previous study [7]. The target phenotype was characterized as a severe physiological status and organ dysfunction (high acute physiology and chronic health evaluation [APACHE II] and sequential organ failure assessment [SOFA] scores), coagulopathy (low platelet count, prolonged PT-INR, low fibrinogen, and extremely high FDP and D-dimer levels), high lactate level, and high mortality.

*Statistical analysis*

We described patient characteristics and clinical course using summary statistics as appropriate. In addition, we reported the number of patients who met the inclusion criteria for the SCARLET trial (cardiovascular and/or respiratory dysfunction, and PT-INR>1.4 and a platelet count in the range from 30 to 150*10^9^/L) to illustrate the difference in the target study population between studies [8]. Cardiovascular dysfuntoin was defined as the use of vasopressor. Respiratory dysfunction was defined as the use of mechanical ventilation.

We derived our prediction model using the JSEPTIC-DIC study and Tohoku Sepsis Registry (derivation cohort) and validated the model using the FORECAST sepsis study (validation cohort). We imputed missing predictors using the random forest method with the *missForest* package [9]. This approach generated single-point estimates by random draws from independent normal distributions centred on conditional means predicted by random forest. Random forest applies bootstrap aggregation of multiple regression trees to reduce the risk of overfitting and combines estimates from many trees. Missingness was imputed using patient characteristics, laboratory data, outcomes, and other covariates, including in-hospital management: age, sex, weight, severity of the sepsis during admission (APACHE II score, each component of SOFA score, systemic inflammatory response syndrome score), comorbidities (cardiac, pulmonary, liver, renal, immunologic), laboratory test results on admission (white blood cells, haemoglobin, PT-INR, FDP, fibrinogen, d-dimer, antithrombin, lactate), treatments (rhTM, antithrombin, protease inhibitors, steroids, immunoglobulins, continuous renal replacement therapy, polymyxin B-immobilized fibre column hemoperfusion therapy) and outcomes (28-day mortality, ICU mortality, and length of hospital stay).

The characteristics of missing data were as reported in our previous study [7]. We did not calculate sample size in advance because we used all available data. The sample size for model development (n=3694, of which 9% had the target phenotype) was enough to ensure precise predictions and minimise overfitting [10].

For model development, we divided the derivation cohort into the training set (70% of the full sample randomly chosen for model development and hyperparameter tuning) and test set (30% of the full sample randomly chosen for internal validation). Using the training set, using log-transformed predictors, we constructed a prediction model with XGBoost. We used the grid search strategy to identify the best combination of hyperparameters using the *ranger* and *caret* packages with 10-fold cross validation.

We measured the prediction performance of the developed model by computing the 1) C statistic (i.e., the area under the receiver operating characteristic [ROC] curve) and 2) prospective prediction results (i.e., sensitivity, specificity, positive predictive value, negative predictive value).

In addition, among patients those who were predicted , we assessed the effect of rhTM on in-hospital and 28-day mortality using a generalised estimating equation to account for patient clustering within hospitals. We assumed an independent correlation structure, and robust standard errors were used to accommodate heteroscedasticity. In the test set of derivation cohort, the adjusted variables were age, sex, comorbidities, and SOFA scores [7]. In the validation cohort, the adjusted variables were age, sex, comorbidities, SOFA scores, and in-hospital management, including renal replacement therapy, and treatment with steroids, intravenous immunoglobulin, antithrombin, and vasopressors [7].

Lastly, All analyses were performed with R statistical software version 3.6.1 (R Foundation for Statistical Computing).

1. Collins GS, Reitsma JB, Altman DG, Moons KG: **Transparent Reporting of a multivariable prediction model for Individual Prognosis or Diagnosis (TRIPOD): the TRIPOD statement**. *Ann Intern Med* 2015, **162**(1):55-63.

2. Hayakawa M, Yamakawa K, Saito S, Uchino S, Kudo D, Iizuka Y, Sanui M, Takimoto K, Mayumi T: **Nationwide registry of sepsis patients in Japan focused on disseminated intravascular coagulation 2011-2013**. *Sci Data* 2018, **5**:180243.

3. Kudo D, Kushimoto S, Miyagawa N, Sato T, Hasegawa M, Ito F, Yamanouchi S, Honda H, Andoh K, Furukawa H *et al*: **The impact of organ dysfunctions on mortality in patients with severe sepsis: A multicenter prospective observational study**. *J Crit Care* 2018, **45**:178-183.

4. Abe T, Ogura H, Shiraishi A, Kushimoto S, Saitoh D, Fujishima S, Mayumi T, Shiino Y, Nakada TA, Tarui T *et al*: **Characteristics, management, and in-hospital mortality among patients with severe sepsis in intensive care units in Japan: the FORECAST study**. *Crit Care* 2018, **22**(1):322.

5. Dellinger RP, Levy MM, Rhodes A, Annane D, Gerlach H, Opal SM, Sevransky JE, Sprung CL, Douglas IS, Jaeschke R *et al*: **Surviving sepsis campaign: international guidelines for management of severe sepsis and septic shock: 2012**. *Crit Care Med* 2013, **41**(2):580-637.

6. Levy MM, Fink MP, Marshall JC, Abraham E, Angus D, Cook D, Cohen J, Opal SM, Vincent JL, Ramsay G *et al*: **2001 SCCM/ESICM/ACCP/ATS/SIS International Sepsis Definitions Conference**. *Crit Care Med* 2003, **31**(4):1250-1256.

7. Kudo D, Goto T, Uchimido R, Hayakawa M, Yamakawa K, Abe T, Shiraishi A, Kushimoto S: **Coagulation phenotypes in sepsis and effects of recombinant human thrombomodulin: an analysis of three multicentre observational studies**. *Crit Care* 2021, **25**(1):114.

8. Vincent JL, Francois B, Zabolotskikh I, Daga MK, Lascarrou JB, Kirov MY, Pettila V, Wittebole X, Meziani F, Mercier E *et al*: **Effect of a Recombinant Human Soluble Thrombomodulin on Mortality in Patients With Sepsis-Associated Coagulopathy: The SCARLET Randomized Clinical Trial**. *JAMA* 2019, **321**(20):1993-2002.

9. Stekhoven DJ, Buhlmann P: **MissForest--non-parametric missing value imputation for mixed-type data**. *Bioinformatics* 2012, **28**(1):112-118.

10. Riley RD, Ensor J, Snell KIE, Harrell FE, Jr., Martin GP, Reitsma JB, Moons KGM, Collins G, van Smeden M: **Calculating the sample size required for developing a clinical prediction model**. *BMJ* 2020, **368**:m441.
