# Supplementary material for: Web-based application for predicting the potential target phenotype for recombinant human thrombomodulin therapy in patients with sepsis: analysis of three multicentre registries
Source: Crit Care. 2022 May 19;26:145. doi: 10.1186/s13054-022-04020-1 (PMC9121613; doi:10.1186/s13054-022-04020-1)
Supplement: Supplementary file 2 — Additional file 2: Table S1. Missingness in predictors and outcome variables. [file 13054_2022_4020_MOESM2_ESM.docx]

**ADDITIONAL FILE 2: TABLE**

**Web-based application for predicting the potential target phenotype for recombinant human thrombomodulin therapy in patients with sepsis: analysis of three multicentre registries**

**Additional file 2: Table S1. Missingness in predictors and outcome variables**

| **Variables** | **Derivation cohort (n = 3694)** | **Validation cohort (n = 1184)** |
| --- | --- | --- |
| Platelets | 84 (2.3%) | 6 (0.5%) |
| PT-INR | 343 (9.3%) | 38 (3.2%) |
| Fibrinogen | 1022 (27.7%) | 226 (19.1%) |
| FDP | 1344 (36.3%) | 376 (31.8%) |
| D-dimer | 1059 (28.6%) | 301 (25.4%) |
| 28-day mortality | 117 (3%) | 0 (0%) |

Abbreviations: FDP, fibrinogen/fibrin degradation product; PT-INR, prothrombin time-international normalised ratio
